# On the road to smart biomaterials for bone research: definitions, concepts, advances, and outlook

**DOI:** 10.1038/s41413-020-00131-z

**Published:** 2021-02-11

**Authors:** Carolina Montoya, Yu Du, Anthony L. Gianforcaro, Santiago Orrego, Maobin Yang, Peter I. Lelkes

**Affiliations:** 1 Department of Oral Health Sciences, Kornberg School of Dentistry, Temple University, Philadelphia, PA 19140 USA; 2 Department of Endodontology, Kornberg School of Dentistry, Temple University, Philadelphia, PA 19140 USA; 3 Guangdong Provincial Key Laboratory of Stomatology, Department of Operative Dentistry and Endodontics, Guanghua School of Stomatology, Affiliated Stomatological Hospital, Sun Yat‐sen University, Guangzhou, Guangdong, China; 4 Bioengineering Department, College of Engineering, Temple University, Philadelphia, PA 19122 USA

**Keywords:** Biological sciences, Bone

## Abstract

The demand for biomaterials that promote the repair, replacement, or restoration of hard and soft tissues continues to grow as the population ages. Traditionally, smart biomaterials have been thought as those that respond to stimuli. However, the continuous evolution of the field warrants a fresh look at the concept of smartness of biomaterials. This review presents a redefinition of the term “Smart Biomaterial” and discusses recent advances in and applications of smart biomaterials for hard tissue restoration and regeneration. To clarify the use of the term “smart biomaterials”, we propose four degrees of smartness according to the level of interaction of the biomaterials with the bio-environment and the biological/cellular responses they elicit, defining these materials as inert, active, responsive, and autonomous. Then, we present an up-to-date survey of applications of smart biomaterials for hard tissues, based on the materials’ responses (external and internal stimuli) and their use as immune-modulatory biomaterials. Finally, we discuss the limitations and obstacles to the translation from basic research (bench) to clinical utilization that is required for the development of clinically relevant applications of these technologies.

## Revisiting the term “smart biomaterials”

**Fig. 1:**
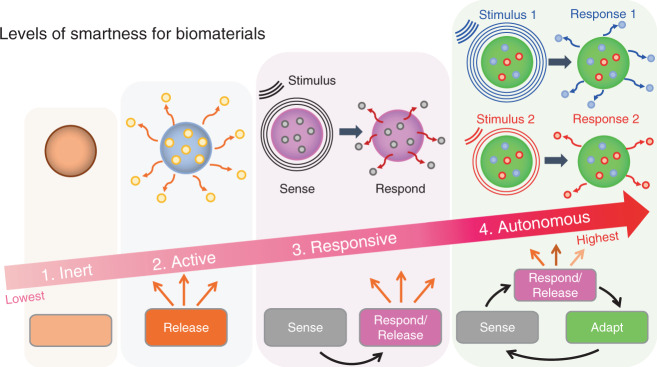
The four increasing levels of smartness for biomaterials include inert, active, responsive, and autonomous. Inert biomaterials offer “merely” biocompatibility and do no harm, i.e., no toxic reaction in/to the body. Active biomaterials offer a one-way, uncontrolled release of therapeutics. Responsive biomaterials can sense specific signals found in the environment or biological processes to then release therapeutics. Autonomous biomaterials can sense a signal, release a specific payload, and adapt their properties to changing conditions to keep providing additional, advanced, and/or alternative forms of therapeutics

Biomaterials have been employed to augment body functions and/or replace damaged tissues for the past several thousand years.^1,2^ Specifically, biomaterials have been instrumental in transforming medicine over the last few decades. Historically, there are three distinct generations of biomaterials which can be labeled as “bioinert”, “biocompatible” and “bioactive”, depending on the degree of their interactions with the body.^3^ The term “Smart Biomaterials” was first coined in 2004,^4^ describing materials “that respond to specific cellular signals”. However, the exponential growth in the last decades of new biomaterials with clever, precise, and highly controlled biofunctionalities warrants a redefinition and clarification of the term. The term “smart” is relative to a particular point in time. Biomaterials that are currently considered “smart” could be considered “dumb” 40 years from now. It is a safe bet to assume that today’s “smart biomaterials” will be “outsmarted” by future innovations. Therefore, in this review, we propose a new classification for smart biomaterials according to their degree (or level) of interaction with their environment and the ensuing biological responses. This classification helps to clarify how smart a biomaterial is. This classification also recognizes the evolution of the concept “smartness” without cementing the definition of what a smart biomaterial is. Hence, it is appropriate to define a level or degree of smartness to help distinguish the materials’ ability to elaborate different sets of biofunctionalities. Thus, defining a scale or degree of smartness will help clarify potential misconceptions, especially for novel biomaterials able to respond to different sources of stimuli. Utilizing control theory as inspiration,^5^ we propose to recognize four levels of smartness for biomaterials, namely inert, active, responsive, and autonomous (Fig. 1). Such classification discerns the various classes of biomaterials according to their degree of interaction with the (bio)environment and, specifically, with biological/cellular processes.

The first and lowest degree of smartness is inert. It is defined as the ability of a biomaterial to be just biocompatible/bioinert, i.e., “to do no harm”, but not to exert any additional biological benefits (Fig. 1). The fact that a material can be used inside the body already elicits a degree of smartness, albeit at the lowest level. Inert biomaterials do not have therapies or bioactive interactions: the biomaterial is accepted by the host without being toxic or generating irritation. For example, Mayan tribes between 350 and 400 CE successfully used jadeite stones to replace teeth.^6^ Different classes of inert synthetic materials including ceramics, polymers, metals, and composites have been proposed for bone applications and approved by the U.S. Food and Drug Administration (FDA). For instance, the classic 316 L stainless steel has been used as metal implants for fracture fixation, and stents; zirconia ceramic for joint replacements and dental implants; poly(methyl methacrylate) polymer for bone cement. While still relevant and necessary, biomaterials that are solely bioinert and biocompatible will no longer be considered sufficient for biomedical applications in the future,^7^ due to significant recent progress in the field of the biomaterials.

The second degree of smartness is *active*. It is defined as the ability of a biomaterial to release a one-way bioactive therapy (i.e., open-loop) (Fig. 1). Active biomaterials are designed to provide a planned one-way interaction with biological processes or with the surrounding environments.^8^ This level of smartness could be analog to the traditional activation of a biological process. For example, *active* dental resin composites have been designed to release antibacterial agents (e.g., Silver-Ag).^9^ The antibacterial therapy inhibits acid-producing bacteria, thus preventing degradation of dental hard tissues and extending the clinical service of dental restorations.^10^ Major limitations of active biomaterials include the limited duration and efficacy of the therapy due to uncontrolled leaching or release of the bioactive compound as a result of their characteristic burst effect. For example, in dentistry, fluoride-releasing composites are prominent antimicrobial treatments.^11^ Most of these systems release fluoride by diffusion. As a result, the antibacterial properties are depleted relatively quickly (<2 years), limiting their long-term performance and leading to the ultimate loss of mechanical integrity.^12^

Active biomaterials have been employed in tissue engineering, for instance, to directly foster hard tissue regeneration through enhanced cell adhesion, proliferation, and differentiation,^13^ or indirectly, by activating the innate immune system to generate regenerative cues via macrophage polarization from an inflammatory (M1) phenotype to reparative/regenerative (M2) phenotype. The latter will orchestrate a distinct set of cellular responses which then lead to the regeneration of tissue.^14^ Active biomaterials have also been utilized for the controlled release of drugs,^15,16^ for the supply of antibacterial,^17,18^ antioxidant and anticancer therapeutics,^19,20^ and for the creation of chemical bonds with surrounding tissues to improve bonding with natural tissues.^21^

**Fig. 2:**
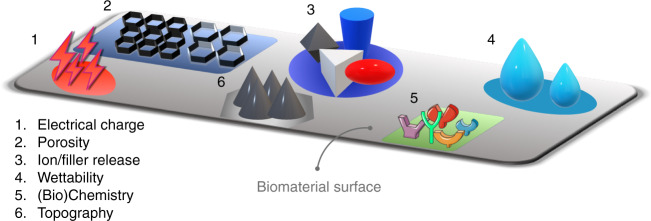
Examples for different surface physicochemical modifications/cues used to improve the degree of smartness on biomaterials

Multifunctional active biomaterials aim to combine the effects of multiple active additives within the same biomaterial for synergistic effects.^22^ For example, a dental resin composite comprised of a matrix of bisphenylglycidyl dimethacrylate and triethylene glycol dimethacrylate embedded with both antibacterial (Ag) and remineralization agents (amorphous calcium phosphate (ACP)) in the form of nano-fillers is able to reduce the impact of acid-producing bacteria and promote mineral formation at the bonded dentin/restoration interface improving the strength and resistance to fatigue of dental restorations.^9^ Common strategies to augment the activity (i.e., increase the degree of smartness) at the host/biomaterial interface include modifications of the biomaterial surface in terms of chemistry, wettability, topography, stiffness, electrical charge, porosity, and leaching of ions, among many others^23–25^ (see Fig. 2). The goal of modifying the biomaterial surface is to create specific chemical and physical environments that offer more favorable, often complex cellular and environmental responses.^26^

The third degree of smartness is *responsive*. It is defined as the ability of a biomaterial to sense a stimulus and react/respond to it by releasing specific therapeutic agents.^27^ It is analog to having a closed-loop feedback control system (Fig. 1). Such biomaterials are usually also coined as stimulus-responsive^28,29^ or bioresponsive.^8,30,31^ Responsive biomaterials will sense/react to particular internal and external signals and then initiate/execute a specific biofunction (e.g., release of a specific therapeutic agent).^32^ These biomaterials are mostly employed as therapeutic platforms for the delivery of precision medications and tissue engineering applications.^33^ For example, poly(ethylene glycol) (PEG) diacrylate hydrogels could sense specific enzymes (e.g., protease) discharged from the surrounding cells to then release growth factors (e.g. FGF-1) for tissue regeneration.^34–36^

**Fig. 3:**
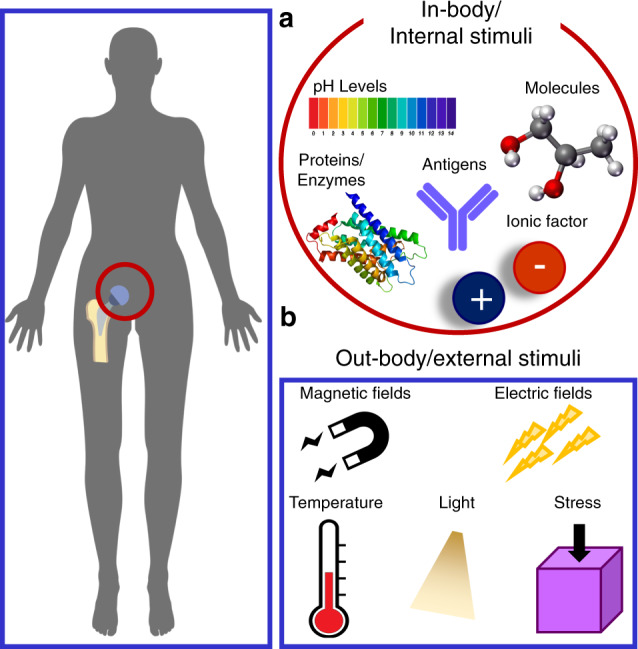
Different types of internal and external stimuli used to enable diverse biofunctionalities of biomaterials. **a** In-body/internal biomaterials respond to signals or stimuli in the immediate vicinity of the implanted biomaterial. Some examples of these signals include enzymes, antigens, proteins, pH levels, and ionic factors. **b** Out-body/external signals are located distally from the biomaterial (e.g., outside the body) and could include electromagnetic fields, mechanical stresses, or temperature changes. Some signals, including temperature, can be found both in-body and out-body

The functionalities of bioresponsive biomaterials can be triggered by different types of stimuli from internal (e.g., in-body) or external (i.e., out-body) sources (see Fig. 3). In-body or internal sources are defined as signals or stimuli found inside the body in the microenvironments in the vicinity of the biomaterial (see Fig. 3a). Out-body or external sources are defined as signals or stimuli found outside the body and not directly in contact with the biomaterial (see Fig. 3b). Internal and external signals are grouped into three major categories: physical, chemical, and biological (or physiological). Internal physical stimuli may include mechanical stress, surface topography, and surface charge, while external physical stimuli comprise light, temperature, electrical and magnetic fields.^37,38^ For example, piezoelectric films (PVDF) are activated by external cyclic loading to form calcium phosphate minerals.^39^ Exposure of osteoblastic cells (MC3T3-E1) to an electric field can produce TGF-β through the calcium/calmodulin pathway for cell proliferation, differentiation, and extracellular matrix (ECM) synthesis leading to transient inflammation and tissue repair.^40^

Chemical stimuli include environmental pH levels, ionic factors, and specific molecules, such as glycoproteins and glucose.^37^ Biological (or physiological) stimuli include enzymes, bioconjugates, antigens, reactive oxygen species (ROS), and other biochemical agents (e.g., viruses or bacteria).^22,33^ For example, to prevent biofilm-related infections of bone implants, exopolysaccharide-degrading enzymes such as glucanohydrolases (dextranase and mutanase) and dispersin B are released only to disrupt the matrix of pathogenic biofilms after the local pH levels turn acidic.^41^ Sophisticated versions of bioresponsive biomaterials offer targeted delivery of therapeutics to specific cells, receptors, or biological processes.^42^ For example, injectable photocrosslinkable gelatin (GelMA) hydrogel microspheres loaded with bone marrow‐derived mesenchymal stem cells (BMSCs) can promote bone regeneration by delivering the appropriate cells and growth factors that help to create a permissive three-dimensional environment for enhanced cell survival and bone growth.^43^ Advances in rational multidisciplinary design and fabrication of biomaterials in combination with advanced knowledge and insight into cellular behavior will accelerate the new biomaterials’ revolution.

The fourth and, currently, the highest degree of smartness is *autonomous*. These futuristic types of biomaterials are considered “self-sufficient”^44^ and can independently adjust their properties and therapeutics in response to changes in the surrounding environments and biological processes^45^ (Fig. 1). Often “living biomaterials” are considered autonomous biomaterials^46^ (not to be confused with biomaterials encapsulating living cells or organisms.^47^) These biomaterials not only deliver targeted/precise therapies after receiving a trigger by an appropriate stimulus, but they interact in sophisticated ways with their surroundings by sensing, responding and adapting to specific signals. Ideally, such technologies can sense a particular disease in its earliest form, communicate its presence outside of the body, and treat different stages before any damage is done.^29,44^ For example, a logic-based peptide hydrogel can operate like a tiny computer system, using inputs from the surrounding microenvironment and decide when to release its therapeutic cargo.^48^ These adaptive biomaterials are designed to mimic nature’s complexity by offering the ability to adapt to the microenvironment.

Biomaterials with this degree of smartness are scarce for now and represent the emergent biomaterial class of the future. An early example of a “living scaffold” was developed such that a cellular-dictated mechanism regulates the presentation of an RGD-peptide depending on cell state, enhancing stem cell differentiation and tissue maturation.^49^ This living scaffold leads to an increase in stem cell survival, resulting in extensive cell differentiation. An autonomous biomaterial will act as a living material with the capability to process information from the environment and change the material’s properties to deliver targeted and dose-controlled therapeutics over an extended time. In other words, autonomous biomaterials become a living, programmable, and reconfigurable independent organism without the need for manual intervention for a wide range of functions.^50^ As it continues to evolve, the field of precision medicine will greatly benefit from autonomous biomaterials by enabling individualized therapeutics from a single device/biomaterial that can adapt to the complexity of individual patients.^51^

In general, the community has recognized active, responsive, and autonomous levels as the current generations of “smart biomaterials”, i.e., biomaterials that elicit a tailored interaction with cellular processes and microenvironments. To clarify the use of the term Smart Biomaterials, we suggest differentiating between the terms “smart biomaterials” and “intelligent biomaterials”. Both terms are often used interchangeably. However, the definitions of “smart” and “intelligent” are different. Smart (or smartness) refers to the acquired ability to apply the prior acquired knowledge (gained), whereas intelligent (intelligence) refers to the innate ability to acquire knowledge (inherent).^52,53^ Commonly, a smart biomaterial cannot have any intelligent functions (e.g., self-calibration, self-diagnose, self-validation), while an intelligent biomaterial cannot be smart.^53^ It is appropriate to clarify each concept for a suitable translation to the biomaterials field and prevent ambiguities, especially in the nascent era of the use of artificial intelligence in the medical field. Hence, it is appropriate to clarify the question whether a biomaterial is or is not smart and how smart it is.^54,55^

### Summary

We propose a new classification for biomaterials according to their degree of smartness as inert, active, responsive, and autonomous. The levels of smartness are defined according to the level of interaction of a given biomaterial with its biological environment (biological/cellular processes). The proposed classification of smart biomaterials ranges from inert materials that can be used inside the body without creating any harm, to autonomous materials capable of sensing, responding, and adapting to changes in the biological environment. Currently, we recognize the levels active, responsive, and autonomous as “smart biomaterials”. Thus, the proposed classification helps clarify how smart a biomaterial is.

## Advanced manufacturing of smart biomaterials

Advanced processing methods are key to manufacturing smart biomaterials. With the use of advanced fabrication techniques, such as additive manufacturing, biomaterials with a lower degree of smartness (i.e., inert) can improve their interactions with the environment and gain some smartness without the need for further treatment and modifications. For example, biocompatible metals like titanium oxides (degree of smartness: inert) can be designed and shaped via 3D printing to yield porous scaffolds with improved capacities for osteointegration and osteogenesis (degree of smartness: active).^56^ Contemporary fabrication methods, such as three-dimensional (3D) and four-dimensional (4D) bioprinting, or electrospinning have gained massive attention due to the possibility of obtaining products of customized shapes and sizes with controlled microstructure (complex shapes), distinct nano-, or micro-topography and a high degree of orientation/alignment.^57^ These methods aim to mimic the shapes and characteristics required to replicate biological tissues in terms of their nano-or microstructures,^58,59^ mechanical properties,^60^ chemistry,^61^ charge,^62^ etc. In addition, the emerging paradigm of precision medicine, which uses individual patient information to tailor clinical therapy for enhanced outcomes^63,64^ requires advanced manufacturing tools to realize biomaterials and devices with the desired properties. Thus, the combination of precision medicine and smart biomaterials is expected to facilitate complex surgeries and efficient delivery of therapeutics.^65^ Given the importance and significant amount of available information on advanced manufacturing of biomaterials, the readers are referred to recent reviews addressing current approaches, limitations, and future perspectives.^65–67^

## Applications of smart biomaterials responding to internal material properties

The active interactions of biomaterials with cells, tissues, and also with biological processes related to osteogenesis and bone repair are governed by diverse materials properties, including topography, mechanical properties, surface chemistry, and charge.

### Topography (porosity, roughness, wettability)

**Fig. 4:**
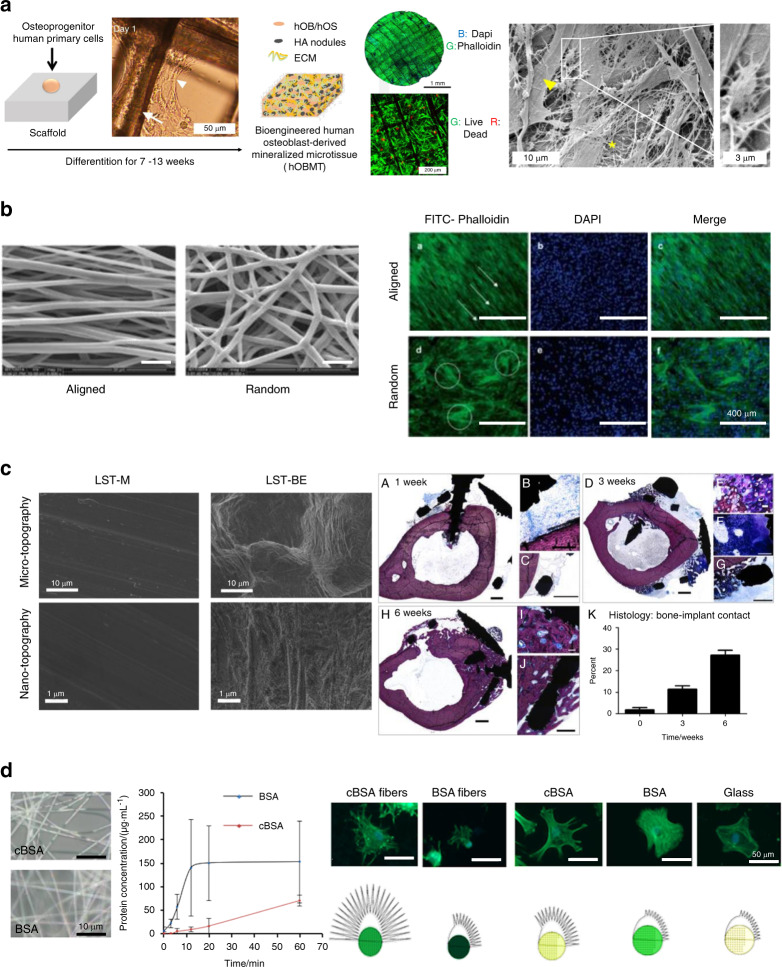
Examples of smart biomaterials responding to intrinsic material properties and its response. **a** 3D printed medical-grade polycaprolactone (mPCL) scaffolds treated with calcium phosphate. Left panel: Schematic showing human primary osteoprogenitor cell seeding on treated mPCL scaffolds. The white arrow and the arrowhead show scaffold fibers and cell organization, respectively. After 7 weeks, the cellular construct leads to the formation of a human osteoblast-derived mineralized microtissue (hOBMT). Middle panel: Staining of the hOBMT shows high cellular organization, strong directional orientation of the actin filaments and >80% cell viability after 10 weeks in culture. Right panel: SEM imaging shows dense ECM deposition (asterisk), osteoblastic cells (arrowhead), and osteocytic cells (insert).^71^ **b** Aligned fibrous polyurethane scaffolds used for culturing annulus fibrosus-derived stem/progenitor stem cells (AFSCs). Left panel: SEM images of the electrospun poly(ether carbonate urethane)-urea (PECUU) scaffolds with aligned and random fibrous microstructures. Right panel: Staining of the AFSCs shows the orientation of the cells along the fibers in the aligned scaffolds while in the random scaffolds, the cells seem randomly oriented.^72^ **c** Subperiosteal titanium-aluminum-vanadium bone onlay fabricated by additive manufacturing (AM) and with post-processing surface treatments. Left panel: Micro and nano topography for disks polished with aluminum oxide sanding paper (LST-M) and grit blasted and acid-etched surfaces (LST-BE). Right panel: Osseointegration was analyzed on surface-modified wrap implants placed around rabbit tibias. After 1 week of implantation, histological sections showed small gaps remaining between the implant screws and bone with new bone and connective tissue (A–C). After 3 weeks, additional bone growth was evident (D–G). At 6 weeks, fully formed bone was present in contact with the inside of the implant (H–J). 6 weeks after implantation, a higher bone-to-implant contact value was observed in histological sections compared to week 1 and 3 and to non-surface-modified constructs (K).^78^ Scale bars for (A–D, H) represent 1 mm, scale bars for (G) and (I) represent 500 μm, scale bar for F represents 200 μm, scale bar for (J) represents 100 μm and scale bar for (E) represents 20 μm. **d** Fibrous scaffold resembling the bone/bone marrow extracellular matrix (ECM) based on bovine serum albumin (BSA). Positive charges in the fibers were introduced via cationization. Left panel: Electrospun fiber morphology was characterized using microscopy (cBSA-cationized; BSA-naive, not cationized). Middle panel: The stability of the fibers against proteases was studied by incubating the fibers in a trypsin/EDTA solution. The cBSA-fibers showed higher stability against proteases compared to the BSA-fibers, as assessed by measuring the timedependent accumulation of BSA in the supernatant. Right panel: MSCs morphology on different substrates (cBSA-fibers, BSA-fibers, cBSA-coated glass, BSA-coated glass and untreated glass). The fluorescent micrographs of cells cultured on cBSA-fibers or cBSA-coated glass showed more and longer protrusions that are related to the regulation of three-dimensional cell migration.^92^ Figures adapted with permission from refs. ^71,72,78,92^

The topography of a biomaterial plays a critical role in initiating and advancing bone regeneration.^68^ An ideal scaffold for bone tissue engineering requires a three-dimensionally interconnected porous structure to support tissue ingrowth.^69,70^ The morphology of the pores on the scaffold surface affects cell spreading and ingrowth (see Fig. 4a, b). In scaffolds with aligned microstructures, cells show better cellular organization when compared with scaffolds where the same microstructures are randomly oriented.^71,72^ For example, Bock et al.^71^ used 3D printing to create a medical-grade polycaprolactone (mPCL) scaffold treated with calcium phosphate to culturing primary human osteoprogenitor cells (Fig. 4a—Left panel). Culturing cells on this scaffold for up to 13 weeks yielded a cellular composite construct with high cellular organization and strong directional actin filaments (Fig. 4a—Middle panel). SEM imaging (Fig. 4a—Right panel) revealed dense ECM deposition, and the presence of osteoblastic and osteocytic cells (insert).^71^ Well-aligned microstructures have also an effect on gene expression and matrix production of collagen. Electrospun scaffolds made of poly(ether carbonate urethane)-urea (PECUU) with aligned and random fibrous microstructures (Fig. 4b—Left panel) were used to culture annulus fibrosus-derived stem cells (AFSCs). Although there was no apparent difference in the attachment or proliferation of cells cultured on aligned or random scaffolds, the AFSCs on the aligned scaffolds were more elongated, better aligned (Fig. 4b—Right panel), and exhibited higher gene expression levels and matrix production of collagen-I and aggrecan.^72^

Some studies suggest that smaller pores improve bone ingrowth by increasing the surface area for increased protein adsorption.^73^ A calcium phosphate cement porous scaffold with pore sizes around 200 μm induced higher serum alkaline phosphate activity and new bone formation in a rabbit cylindrical bone defect model at the early stages of 4 and 12 weeks, when compared to scaffolds with pore sizes of 350–450 μm or 450–600 μm.^74^ Further, polyglycerol sebacate membranes manufactured by salt-leaching with a pore size of ~25 μm provided less cell penetration and membrane degradation than similar membranes with a pore size of 53 μm, which had some obvious advantages, such as increased blood clotting and enhanced generation of new bone in 4–12 weeks during the repair of rabbit tibia defects.^75^ More recently, our group has developed a bilayered poly(d,l-lactide-co-glycolide) (PLGA) scaffold by the diffusion-induced phase separation technique, with one side containing open pores (~45 μm in diameter and with higher roughness ~1.39 μm), while the other side contained closed pores (<5 μm in diameter and with lower roughness ~0.31 μm). Human dental pulp stem cells (DPSCs) penetrated into the larger pores through the channels of the open side, while on the closed side the cells rather spread on the surface, proliferated, and underwent spontaneous osteogenic differentiation.^76^ Other studies indicated that scaffolds with pore sizes around 250 μm might encourage fibrous tissue ingrowth, leading to poor bone repair.^77^

3D-printed scaffolds, comprised of nanocomposite polydopamine‐laced hydroxyapatite (HAp) collagen calcium silicate (HCCS‐PDA) with 500 μm pores, induced more bone regeneration than the same scaffolds with 250 μm pores group, as confirmed by greater bone volume, greater coverage of defect area, and new bone formation when seeded with rat MSCs for repairing surgically created critical‐sized defects. HCCS-PDA scaffolds with smaller pores disintegrated during implantation due to their weaker mechanical stability.^59^ The controversy over the relationship between scaffold pore size and bone regeneration capability may be caused by different laboratories using different cell types, material resources, and scaffold fabrication methods.

Other characteristics of the surface topography of a given biomaterial scaffold include roughness, wettability, and crystallinity. For example, changes in the roughness at the microscale and nanoscale of titanium-aluminum-vanadium implants fabricated using additive manufacturing with postprocessing surface treatments such as polishing with aluminum oxide sanding paper (LST-M) or grit blasted and acid-etched surfaces (LST-BE) (Fig. 4c—Left panel) showed improved osseointegration (measured via bone-to-implant contact) in wrap implants placed around rabbit tibias compared to non-surface-modified constructs^78^ (Fig. 4c—Right panel). After 1 week of implantation, modified constructs showed only small gaps between the implant screws and bone, with evidence for de novo formation of bone and connective tissue. After 6 weeks, fully formed bone was observed and a higher bone-to-implant contact value was observed in histological sections compared to week 1 and 3 and to non-surface-modified constructs.^78^

The roughness of nano-grooved Ti-coated cylindrical epoxy resin implants significantly increased bone volume compared to conventional implants in the rat femur. Specifically, implants with larger nanogrooves (groove width/ridge width/depth: 200/800/70 nm) showed improved osteoinductivity than those with smaller nanogrooves (groove width/ridge width/depth 150/150/50 nm). Because the nanogroove patterns contain both ridges and grooves, the results indicated that the osteoinductivity effect was related more to the ridge areas rather than the groove areas.^79^

In another study calcium silicate was modified with deionized water using hydrothermal treatment to obtain modified calcium silicate (NT-CS) with nanoscale surface topography. Compared to the non-modified calcium silicate with its rough surface, NT-CS was more hydrophilic and exhibited higher crystallinity, increasing cell spreading, and augmenting integrin β-1 expression and collagen secretion of cultured MSCs. Moreover, supplementing NT-CS with strontium ions significantly enhanced ALP activity and calcium deposition, as well as the expression of calcium sensitive receptor, and BMP2, bone sialoprotein (BSP), osteopontin (OPN), osteocalcin (OCN), and osteoprotegerin, while inhibiting the expression of interleukin 6 and receptor activator of nuclear factor kappa-Β ligand, thus promoting MSCs differentiation towards osteoblasts.^80^

Interestingly, chondrocytes maintained their phenotype when cultured on a dense poly-L/D-lactide acid (PLDLA) scaffold. By contrast, the expression of chondrogenic markers SRY-Box Transcription Factor 9 (SOX-9), collagen type II (Col II), and Aggrecan was reduced when the cells were cultured on a nanofibrous PLDLA scaffold, even though these two scaffolds had similar porosities and pore sizes.^81^ Increased surface area and hydrophilicity of the nanofibrous scaffold may have promoted cell–matrix adhesion while decreasing cell–cell contracts, while the nanofibrous scaffold promoted MSC osteogenic differentiation by enhancing the expression of runt-related transcription factor 2 (RUNX2), BSP, and OPN.^81^

### Mechanical properties (stiffness, storage modulus)

Besides changing the basic structural properties of the scaffolds (such as morphology, topography, porosity, and pore size), manipulating substrate stiffness to mimic the natural 3D microenvironment will also impact osteogenic vs. chondrogenic fate decision. Chen et al.^82^ generated scaffolds with the same 3D microstructure of bone but with various stiffnesses (between 13 and 38 kPa) by coating decellularized cancellous bone with a mixture of collagen and HAp in different proportions. Bone marrow-derived mesenchymal stem cells (BMSCs) cultured for 2–3 weeks on these scaffolds in vitro expressed higher levels of OPN and OCN. Importantly, after subcutaneous implantation into rats for 6 months, only the stiffer scaffolds also recruited endothelial cells with high levels of CD34 expression and showed evidence for angiogenesis in the implanted constructs.^82^

The mechanical strength of nanofibrous gelatin (NF-gelatin) scaffolds can be controlled by varying the time and degree of crosslinking. DPSCs cultured on NF-gelatin scaffolds with a high-stiffness (18 kPa) displayed a more organized cytoskeleton (evidenced by F-actin filaments stained by phalloidin 546) and larger spreading areas than cells maintained on NF-gelatin scaffolds with a low stiffness (0.9 kPa). The NF-gelatin scaffold with the higher stiffness facilitated osteogenic/odontogenic DPSC differentiation (expression of collagen type I (Col I), OCN, dentin matrix acidic phosphoprotein 1, ALP, and dentin sialophosphoprotein), while a low-stiffness NF-gelatin scaffold promoted the expression of thyrotropin-releasing hormone degrading enzyme and syndecan3, which are known to be highly expressed in natural pulp tissue. When the low and high-stiffness NF-gelatin scaffolds were combined into a single composite, biphasic scaffold, the biomimetic scaffold was able to regenerate the complete pulpodentin-like complex after subcutaneous implantation into a mouse model.^83^ In the same vein, the stiffness of a transglutaminase cross-linked gelatin (TG-Gel) scaffold was manipulated by controlling the concentration of gelatin (3%, 6%, 9%), with resulting yield strengths of 1.5, 13, and 32 kPa, respectively. The stiffer TG-Gels facilitated more focal contact formation, higher ALP activity, and induced mineralized nodules in cultured C2C12 myoblasts.^84^

The storage modulus of a 2D substrate and a 3D hydrogel not only influences the activities of single cells but also affects multicellular microspheres (organoids) suspended in the hydrogel. In a recent study, Zigon-Branc et al. ^85^ encapsulated adipose stem cell (ASC) organoids in gelatin-based hydrogels of different stiffnesses (storage moduli between 540 and 7 260 Pa). When maintained in a chondrogenic induction medium, all cultures expressed high levels of chondrogenic markers (Sox-9, ACAN, COL2A1). However, the softer hydrogels were more efficient in promoting the formation of more mature, Alcian blue-stained cartilage. Interestingly, softer hydrogels could also induce spontaneous in vitro chondrogenic differentiation of ASC organoids in the absence of chondrogenic induction medium.^85^

### Surface charge

Negatively charged surfaces, while generally repellant to cells, efficiently adsorb serum proteins to promote secondary cell adhesion.^86^ For example, graphene with negative charges attracts DNA and RNA, which is beneficial for tissue engineering.^87^ A reduced graphene oxide (GO)-coated biphasic calcium phosphate (BCP) bone graft with a surface potential at −14.43 mV increased the area of new bone formation in a rat cranial defect model when compared to the pure BCP.^88^ The epigallocatechin gallate (EGCG) modification converted the positive surface potential of a gelatin sponge (+0.24 mV) to a negative surface potential (−0.54 mV), which lead to enhanced cell adhesion and calcium phosphate precipitation, and thus increased bone formation in a rat congenital cleft-jaw defect model.^89^

Positively charged surfaces usually favor cell adhesion through electrostatic interactions with the negatively charged cell membranes. For example, poly(hydroxyethyl methacrylate) (pHEMA) with a high positive charge (+11 mV) had no significant effect on EphB4 activation or MSCs differentiation, as inferred from the low level of expression of osteogenic markers, including RUNX2, OCN, BSP, and OPN. Conversely, the pHEMA with a lower positive charge (+3 mV) promoted the phosphorylation of EphB4 and led to efficient osteogenic MSC differentiation.^90^ In comparing the behavior of MSC cultured on monolayers self-assembled from different alkanethiol solutions, Hao et al. ^91^ found that fewer cells adhered to surfaces with a low (–COOH, –CH_3_, –PO_3_H_2_) or moderate (–OH, –OEG) iso-electric point (IEP) than to surfaces with higher IEP values (–NH_2_).^91^

### Scaffold chemistry (ions, compounds)

The level of smartness of inert biomaterials can be increased by incorporating therapeutically beneficial chemical and bioactive compounds, including ions and growth factors. For example, Raic et al.^92^ fabricated a positively charged fibrous scaffold based on bovine serum albumin (BSA) to mimic the bone/bone marrow ECM (Fig. 4d—Left panel). Positive charges introduced via cationization support the stability of the scaffold in cell culture and acted as nucleation points for mineralization during osteogenesis.^92^ MSCs cells cultured on cationized BSA-fibers revealed enhanced focal adhesion formation, more and longer protrusions that are related to the regulation of three-dimensional cell migration and improved osteogenic differentiation^92^ (Fig. 4d—Right panel).

Doping β-tricalcium phosphate (β-TCP) with metal ions (Ti, Mg, Zn) yielded enhanced bone formation in the rabbit femoral condyle defect. Due to the superior osteogenic effect of Ti, a 5%Ti-β-TCP composite scaffold exhibited the highest capacity to enhance bone formation, as compared to 5% Zn-β-TCP, 5% Mg-β-TCP, and β-TCP alone.^93^ Vieira et al.^94^ synthesized self-mineralizing, calcium-enriched methacrylated gellan gum (GG-MA) hydrogel beads, which, when placed in simulated body fluid, were spontaneously covered with a white mineral layer exhibiting the typical cauliflower-like morphology of HA. The thickness of the mineralized HA layer increased over 8 weeks in vitro. When implanted subcutaneously in CD1 male mice for up to 8 weeks, the beads were completely calcified without any signs of an inflammatory reaction.^94^

As a potential molecule for promoting bone regeneration, specifically for enhancing muscle-bone connectivity, Irisin, a skeletal muscle-derived cytokine, loaded into silk/calcium silicate/sodium alginate composite hydrogels, upregulated the expression of several osteogenic markers containing RUNX2, ALP, BMP2, Osterix, OCN, and OPN in cultured BMSCs and improved the repair of calvarial defects in a rat model in vivo.^95^ Sinapic acid is a plant-derived phenolic compound with potentially beneficial effects for bone regeneration. Electrospun polycaprolactone (PCL) fibrous scaffolds, containing chitosan nanoparticles loaded with sinapic acid, promoted osteoblast differentiation in vitro and accelerated bone regeneration in vivo.^96^

Another strategy to increase the degree of smartness from inert to active of biomaterial-based drug delivery systems entails changing the mode of chemical delivery. In active delivery, the drug release is triggered by environmental stimuli such as temperature,^97^ pH change,^98^ chemical and redox reactions,^99^ and enzymes,^100^ or by external stimuli such as electric or magnetic field, light, etc. Active drug delivery relies on the drug diffusing through the carrier matrix to reach the surrounding medium.^101,102^ Due to the constant evolution and significant amount of information available regarding drug delivery systems for hard tissue regeneration, readers are referred to recent excellent reviews addressing this subject matter.^103–105^

### Summary

Different biomaterial properties and characteristics such as porosity, roughness, chemistry, surface charge, and mechanical properties drive specific interactions with bone cells. A variety of in vitro and in vivo model systems have been used to study materials with characteristics that can mimic a more realistic and suitable environment for promoting cell functions and improve bone modeling and remodeling.

## Applications of smart biomaterials responding to external stimuli

Different types of stimuli can trigger the release of therapeutics or induce changes in the properties of biomaterials to improve their interaction with cells and biological processes for enhanced therapeutic outcomes.

### Piezoelectric materials

Piezoelectric materials can either convert mechanical stress into electrical charges (direct piezoelectricity) or electrical charges into mechanical signals (converse piezoelectricity).^106^ Endogenous direct electric currents are amongst the fundamental biosignals affecting development, regeneration, and wound healing.^107^ Tissues like bone, cartilage, dentin, and tendon can display direct piezoelectric properties.^40^ This effect is likely due to the quasi-hexagonal symmetry of the collagen structure at the nanoscale, and may significantly contribute to the mechanoelectrical transduction mechanisms accompanying bone remodeling.^108^ Potential strategies for mechano-electrically-driven bone regeneration based on piezoelectric biomaterials can be divided into two major categories: piezoelectric polymers and piezoelectric ceramics.^40^

One popular piezoelectric polymer with applications in bone regenerative engineering is polyvinylidene fluoride (PVDF) due to its flexibility and biocompatibility.^109^ 3D scaffolds from PVDF nanofibers and containing additives like GO and EGCG seem to promote osteogenesis.^110^ Osteogenesis-related genes and proteins, such as RUNX2, Col I, and Osteonectin were significantly upregulated in human induced pluripotent stem cells (iPSCs) seeded on 3D scaffolds made of PVDF nanofibers as compared to iPSCs seeded on 2D PVDF films.^110^ When osteoblasts were grown on oxygen plasma-treated permanently hydrophilic nanofibrous PVDF scaffolds, cells demonstrated a higher degree of cell spreading and cell activity, as indicated by the increase in intracellular calcium levels, compared to the same cells grown on non-treated PVDF scaffolds.^111^ PVDF nanofibers modified with GO (5 mg·mL^−1^) promoted osteogenesis in iPSCs culture as inferred from the enhanced expression of RUNX2 and OCN on day 21.^112^

Composite nanofibers comprised of PVDF and polyhedral oligomeric silsesquioxane-epigallocatechin gallate also promoted mineralization of osteoblasts.^113^ Interestingly, the chondrogenic vs. osteogenic differentiation-inducing capacities of a 3D fibrous scaffold, electrospun from poly(vinylidenefluoride-co-trifluoroethylene) (PVDF-TrFE) varied depending on the electric field strength generated by dynamic compression: Exposure of BMSCs to a low electric field (20 mV·mm^−^^1^) promoted the expression/upregulation of chondrogenic differentiation markers, including Col II and Sox9. By contrast, exposure to a high electric field (1 V·mm^−1^) promoted osteogenic differentiation with increased expression of osteogenic markers, such as Col I, ALP, and OCN.^114^ Another study used PVDF-TrFE membranes to study the relationship between the surface electrical potential of the membranes and their osteogenic properties. The membrane surface potential can be regulated by increasing the β-phase content: a membrane with a surface potential of −53 mV showed stronger osteogenic properties than a membrane with −78 mV, as evidenced by enhanced MSC osteogenesis in vitro, and leading to rapid bone regeneration and mature bone structure formation in an in vivo rat calvarial defect model.^115^

Piezoelectric ceramics, such as potassium sodium niobite (KNN), have also been studied in the context of bone tissue engineering/regeneration. KNN with a piezoelectric constant of ~93 pC/N and relative density of ~93% enhanced bovine BSA protein adsorption and promoted osteoblast cell proliferation as compared to non-polarized surfaces.^116^ Application of microscale piezoelectric zones to KNN ceramic surfaces significantly enhanced the expression of Col I, ALP, RUNX2, and OPN of cultured MSCs in vitro, and also promoted bone repair in a rabbit femoral condylar defect model in vivo.^117^

### Magnetic biomaterials

Magnetic stimulation by static magnets or dynamic electromagnetic fields can enhance the healing of bone fractures and promote bone formation.^118,119^ Magnetic nanoparticles (MNPs), such as magnetite (Fe_3_O_4_) and maghemite (Fe_2_O_3_) are clinically approved metal oxides due to their unique property of superparamagnetism. MNPs are often added to biocompatible materials to be guided to specific tissues (magnetic targeting). Although MNPs hold great potential in biomedical applications such as magnetic resonance imaging, drug delivery, and hyperthermia, safety considerations related to superparamagnetism should be always addressed. For example, iron overload caused by MNPs may be cytotoxic, due to the production of intracellular ROS.^120^

MNPs themselves can be considered as a single magnetic domain in promoting osteogenesis/chondrogenesis without an external magnetic field (EMF) stimulus. Magnetic nanofibrous scaffolds fabricated by PCL doped with Fe_3_O_4_-MNPs promoted the adhesion, penetration, and the expression of MSCs osteogenic markers such as BSP and OPN.^121^ An in vivo study showed that chitosan/collagen/Fe_3_O_4_/nano-HAp scaffolds enhanced osteogenic differentiation of MSCs and facilitated bone growth in a rat calvarial defect model.^122^ Another study incorporated MNPs into a hybrid magnetic hydrogel (MagGel), containing Col II, hyaluronic acid, and PEG. BMSCs cultured on MagGels showed adhesion densities and cell morphologies similar to those of BMSCs cultured on the hybrid gel without MNPs, suggesting that the MNPs did not affect BMSCs morphology and adhesion. The authors hypothesized that the ingested nanoparticles may be broken down in the lysosomes and excreted via exocytosis, therefore not impacting BMSCs morphology and adhesion.^123^

Responding to an EMF generated by permanent magnets, MNPs support bone/cartilage formation. After incorporating MNPs (Fe_3_O_4_) into the mineralized collagen coatings of a titanium substrate, the EMF produced by a permanent magnet stimulated osteogenic differentiation in cultured osteoblasts.^124^ When ASCs and/or human tendon-derived cells were encapsulated in hydrogels containing both platelet lysate (PL) and Fe_3_O_4_ MNPs, the EMF from a commercial magnefect nano device modulated the swelling, degradation of the hydrogels and the release of PL-derived growth factors, resulting in altered cell morphology and the expression of tissue-specific marker genes, thus promoting the synthesis of tendon and bone-like matrices respectively.^125^

**Fig. 5:**
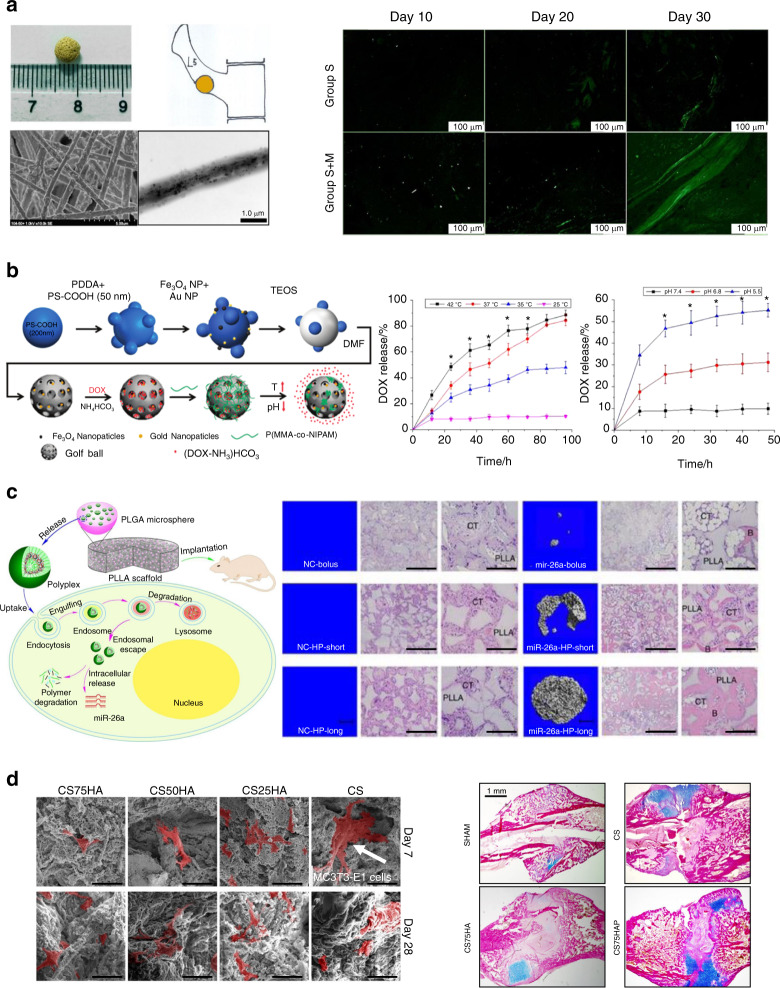
Examples of smart biomaterials responding to extrinsic stimuli and its response to promote bone regeneration. **a** Magnetic biomaterials—nanofibrous composite scaffold composed of super-paramagnetic γ-Fe2O3 nanoparticles, hydroxyapatite nanoparticles (nHA), and poly lactide acid (PLA). Left panel: An ~ 5 mm scaffolds were implanted in the defect of transverse process of L5 of rabbits. SEM and TEM images show randomly tangled nanofibers with diameters between 300 and 1 000 nm. Right panel: After 30 days of implantation, a higher collagen deposition was found for the scaffold tested under a magnetic field (S + M) compared to the unstimulated group (S).^126^ **b** Magnetic biomaterials—magnetic and gold nanoparticles embedded silica nanoshuttles (MGNSs) with nanopores on their surface. Left panel: The MGNSs are loaded with fluorescently labeled doxorubicin (DOX) and encapsulated in a thermo- and pH-sensitive polymer to enable the controlled release of the drug into composite human tissues (i.e. bone). Right panel: Profiles of DOX release at different temperatures for 100 h and at different pH conditions for 50 h.^152^ **c** Enzyme-responsive materials—PLLA nanofibrous scaffold containing a hyperbranched polymer with microRNAs polyplexes encapsulated into PLGA microspheres to regenerate critical-sized bone defects. Left panel: The miRNA is released after enzymatic polymer degradation promoting regulation of gene expression. Right panel: A subcutaneous implantation model was used to evaluate the efficiency of delivery of miR-26a in three groups (bolus, short-term, and long-term delivery). There was no appreciable new bone in the negative groups (NC). New bone volume was found for all miR-26a delivery groups, being highest for the long-term delivery group, followed by the short-term and bolus group.^155^ Scale bars, 1 mm (in microCT images- left images), 500 μm [in haematoxylin and eosin (H&E) images- center images], 200 μm (in higher-magnification H&E images at far right). **d** Enzyme-responsive materials:- Guanosine 5′-diphosphate-cross-linked chitosan scaffolds (CS) with different amounts of Hydroxyapatite (HAp) (75%—CS75HA, 50%—CS50HA, and 25%—CS25HA) were tested with or without pyrophosphatase activity. Left panel: When cultured with MC3T3-E1 cells, a higher number of cells on day 28 was observed compared to day 7, indicating cell proliferation in the scaffolds. For both times points and in all scaffolds the cells showed a spread morphology and well-organized F-actin filaments. Magnification: 5 000X. Right panel: The in vivo osteogenic properties of the scaffolds was tested in a murine model of rod-fixation tibia fracture surgery. After 17-days of implantation, an increased callus formation at the fracture site was found for the scaffold with pyrophosphatase and HAp (CS75HAP) by comparison to the control scaffolds lacking both pyrophosphatase and HAp (CS), or pyrophosphatase alone (CS75HA).^169^ Figures adapted from refs. ^126,152,155,169^ with permission

A 5 mm nanofibrous composite scaffold composed of γ-Fe2O3 MNPs, hydroxyapatite nanoparticles (nHA) and poly(lactic acid) (PLA) was implanted in the defect of transverse process of L5 of rabbits as shown in Fig. 5a (Left panel). An external static magnetic field was applied to validate in-vivo osteogenesis enhancement. The super-paramagnetic nanofibrous scaffold accelerates bone tissue regeneration under the EMF compared with the scaffolds without the external activation.^126^ After 30 days of implantation, higher collagen deposition was found for the scaffold tested under a magnetic field (S + M)^126^ (see Fig. 5a—Right panel). After 110 days of implantation, the stimulated scaffold (S + M) was completely absorbed while small amounts of scaffold were observed for the unstimulated group (S). MNPs may also play an important role in regulating inflammation. A protein corona analysis in a rat model indicated that the presence of MNPs in HAp scaffolds suppressed chronic inflammatory responses while promoting acute inflammatory responses, which in turn led to the recruitment of CD4^+^ T-lymphocytes, remodeling of the ECM, and acceleration of bone healing.^127^

Enhanced scaffold biodegradation has usually been accompanied by accelerated bone formation. For example, one study used an osteoconductive magnetic 3D scaffold [Fe^2+^ doped nano-hydroxyapatite-Alginate-Gelatin (AGHFe1)] to support bone tissue regeneration. Two modifications were done on AGHFe1 including the scaffold loaded with recombinant human bone morphogenetic protein-2 (rhBMP-2), named AGHFe2, and the scaffold with the degradation rate adjusted, named AGHFe3. When implanted into a rat cranial defect, the faster degradation rate of AGHFe3 scaffolds yielded enhanced bone formation in comparison to scaffolds that degraded slower (AGHFe2), as inferred from the increased expression of ALP and bone marker genes, as well as increased mineral deposition.^128^

### Shape memory biomaterials

Shape memory materials (SMMs) can recover their original shape from a significant and seemingly plastic deformation when a particular stimulus is applied. Currently, the family of SMMs includes shape memory alloys (SMAs), shape memory polymers (SMPs), and shape memory hybrids.^129^ Nickel-titanium alloy cycles between the deformable martensite and the “memory” austenite configurations. These changes are induced by heating or cooling, and in some temperature ranges by mechanical loading or unloading.^130^ Due to its special physicochemical properties, NiTi can be used as a functional implant that can provide a continuous force to the bone. In one animal study using a rat femur model, pre-shaped, curved NiTi nails were implanted intramedullary in the cooled martensite form. Upon warming to body temperature and regaining their austenite form, the nails provided a bending force to help form the angle between the distal articular surface and the long axis of the femur during bone regeneration.^131^ Frequently, NiTi SMAs are manipulated to the desired shape in vitro and then applied in vivo, e.g., as bone distractor.^132–134^ In a recent study, a NiTi-SMA plate was heated by electromagnetic stimulation, yielding higher material stiffness, which in turn resulted in increased fixation stiffness and resulted in better bone healing in a rabbit osteotomy model.^135^ Importantly, NiTi SMAs have already been used clinically, including in patients with cleft lip and palate,^136^ in patients undergoing midfoot or hindfoot arthrodesis,^136^ in treating adolescent idiopathic scoliosis,^137^ etc.

In contrast to SMAs, SMPs are lightweight, easy to fabricate, more elastic, and may be biodegradable. SMPs provide better contacts between the scaffolds and the surrounding bone tissue. Most of the SMPs are thermo-responsive; they are malleable at temperatures above their transition temperatures while cooling locks the scaffolds into their new temporary shapes.^130^ A PCL-based SMP scaffold coated with polydopamine enhanced osteoblast adhesion, proliferation, and osteogenic gene expression.^138^ A fibrous PCL-PDMS (polydimethylsiloxane) scaffold also promoted proliferation and enhanced osteogenic ALP activity of cultured osteoblasts.^139^

Another study compared the capability of two scaffolds to support adipogenesis: the first one was a foam SMP scaffold, which was produced by crosslinking tert-butyl and acrylate:butyl-acrylate using a modified porogen-leaching method and then coated with polydopamine. The second scaffold was a fibrous SMP scaffold produced by electrospinning a custom-synthesized thermoplastic polyurethane. ASCs showed comparable osteogenic differentiation on both SMP scaffolds over a 23-day culture period.^140^ In a follow-up study with the same SMPs scaffold, irrigation with 45 °C warm saline during surgery expanded the scaffold in a controlled manner and integrated with native bone in a mouse segmental defect model.^141^

Similarly, a polyurethane/HAp-based SMP scaffold was expanded by irrigation with 40 °C saline and provided a tight fit in a rabbit femoral bone defect model, thus facilitating bone regeneration in vivo.^132^ SMMs made of natural polymers can also change their shapes upon hydration. For example, scaffolds made of native collagen or denatured collagen (gelatin) display shape memory properties and can immediately recover their original shape upon rehydration.^142^ However, native collagen scaffolds better sustain chondrocyte proliferation, differentiation, and function than denatured collagen scaffolds, probably due to their triple-helical structure.^142^

### pH-/thermo-responsive biomaterials

Amongst the stimulus-responsive SMPs, thermo-responsive hydrogels exhibit a sol-to-gel transformation, which is triggered by proper physiological stimuli, such as exposure to body temperature at 37 °C or pH at 7.4. During the sol–gel transformation process, cells and growth factors can be incorporated into the sol in vitro, which then can be delivered to the desired physiological environment in a minimally invasive way by injection and finally reside in the target tissues as a 3D gel scaffold with controlled-release properties.^143^ Thermosensitive hydrogels have been employed for several decades in bone tissue engineering. For example, a composite hydrogel made of thermosensitive poly(N-isopropylacrylamide) and combined with gelatin was applied as an injectable delivery vehicle of MSCs for the repair of a rat cranial defect.^144^

A hyaluronic acid-g-chitosan-g-poly(N-isopropyl acrylamide) (HA-CPN) copolymer hydrogel was used as a cell carrier for inducing osteogenic differentiation in cultured MSCs and ectopic bone formation in a mouse model in vivo.^145^ Furthermore, an HA-CPN hydrogel, containing platelet-rich plasma and BCP as an injectable cell carrier for osteogenic ASCs, promoted osteogenesis both in vitro and vivo.^146^ Similarly, a methylcellulose hydrogel containing calcium phosphate nanoparticles showed great potential for bone tissue regeneration.^147^

Finally, simvastatin‐loaded PLGA-PEG hydrogel promoted osteoblast differentiation.^148^ The sol-to-gel transformation can also be triggered by changes in the pH. A pH-sensitive composite hydrogel composed of sulfamethazine oligomers combined with a thermosensitive poly(ɛ-caprolactone-co-lactide)–PEG–poly(ɛ-caprolactone-co-lactide) formed an injectable sol (pH 8.0 and 37 °C) which then turned into a stable gel under physiological conditions (pH 7.4 and 37 °C). When incorporating BMP2, this hydrogel more efficiently promoted MSCs differentiation than the same hydrogel without protein.^149^ A composite hydrogel combining carboxymethyl chitosan and ACP nanoparticles assembled in a pH-triggered, controlled fashion. This composite hydrogel showed favorable biocompatibility and osteoinductivity and also amplified the BMP9-induced osteogenic differentiation of MSCs.^150^

More recent studies demonstrated that some biomaterials can respond to the changing pH in an inflammatory environment. For example, when loaded with the broad-spectrum antibiotic agent levofloxacin, a nanocomposite ceramic scaffold composed of nanocrystalline apatite and mesostructured SiO_2_–CaO–P_2_O_5_ glass wall (MGHA) displayed sustained drug delivery at physiological pH (pH 7.4). The rate of drug delivery increased notably when the pH decreased to values characteristically associated with bone infection (pH 6.7 and pH 5.5), which renders this system beneficial for preventing bone infection.^151^

A similar example of drug delivery, activated by thermal-pH changes, can be seen in Fig. 5b. Hollow silica nanoshuttles, made of hybrid materials, such as silica, gold, and iron oxide nanoparticles and loaded with fluorescently labeled doxorubicin (DOX), were encapsulated in a thermo- and pH-sensitive polymer to enable the controlled release of the drug into diverse human tissues such as bone, cartilage, tendon, bone marrow, and brain for highly efficient personalized medicine applications.^152^ A scheme for the synthesis of gold and MNPs embedded in hollow silica golf balls (MGNS), DOX loading, and P(MAA-co-NIPAM) coating is presented in Fig. 5b (Left panel). The MGNS are enclosed in a heat and pH-sensitive polymer P(NIPAM-co-MAA). After the polymer is externally stimulated (temperature or pH stimuli) the DOX is released.^152^ The releasing efficiency at different temperatures and pH are presented in Fig. 5b (Right panel).

### Enzyme-responsive biomaterials

Enzymes play key roles in diverse biological processes, such as growth, blood coagulation, healing, breathing, digestion, etc. An imbalance in enzyme expression and/or activity can lead to serious diseases, such as cancer, cardiovascular disorders, inflammation, degenerative arthritis, among others.^153^ Enzyme-responsive materials (ERMs) are triggered by selective catalytic actions of specific enzymes.^154^ For example, Zhang et al. ^155^ created an implanted poly(L-lactic acid) (PLLA) nanofibrous scaffold containing a hyperbranched polymer (HP)/miRNA polyplexes encapsulated into PLGA microspheres to regenerate critical-sized bone defects (see Fig. 5c—Left panel). After enzymatic degradation of the polymer, the miRNA is released to regulate local gene expression and promoting new bone formation.^155^ A murine subcutaneous implantation model was used to evaluate the efficiency of delivery of miR-26a into cells at three different times (bolus, short-term, and long-term delivery) and the ability to upregulate the expression of osteogenic factors, increasing new bone volume. After 8 weeks of implantation, new bone formation was found for all miR-26a delivery groups compared to the negative control (NC), the new bone volume being the largest for the long-term delivery group, followed by the short-term application and the bolus groups^155^ (see Fig. 5c—Right panel). The main advantage of ERMs is that their activation does not require external stimuli since the enzymatic changes are within the biological system and the enzyme activity is controlled by changes in the biological environment itself, showing high efficacy in enzymatic catalysis.^156,157^ In contrast to some other smart biomaterials, ERMs, do not require external stimuli, such as temperature or pH that could affect other components of the environment.^158^ For example, during an inflammatory event—a highly regulated biological response—, an enzyme-responsive scaffold could help modulate the process of tissue healing through its different stages without the need of an external stimulus. Currently, typical enzymatic triggers include proteases such as matrix metalloproteinases (MMPs),^159,160^ phosphatases such as ALP,^161,162^ redox enzymes such as glucose oxidase^163,164^ and glycosidases such as hyaluronidase.^33^ However, ERMs have a major limitation in that the response to an enzymatic stimulus can in some cases depend on the age of the host.^165^

Recently, ERMs have also been used for the delivery of growth factors to accelerate the healing of bone fractures^159^ and scaffold degradation,^166^ for enhanced delivery of drugs^153,161^ and cells,^167^ or for the generation of implants with multifunctional capabilities (i.e., antibacterial and tissue regeneration).^168^ For example, degradation of chitosan scaffolds generates inorganic pyrophosphate (PPase),—an inhibitor of physiologic mineralization—. However, with the addition of the enzyme pyrophosphatase (PPase), the scaffold breaks down PP_i_ into two phosphate ions, which are essential for the mineralization of the ECM in a bone healing process^169^ (see Fig. 5d). A guanosine 5′-diphosphate-cross-linked chitosan scaffold with encapsulated HA and PPase in differences proportions showed to be suitable for the MC3T3-E1 cells’ proliferation, evidenced by a spread morphology and well-organized F-actin filaments (see Fig. 5d—Left panel). During an in-vivo tests, the same scaffold with 75% of HAp and PPase showed increased callus formation at the fracture site in comparison to scaffolds without HAp or PPase^169^ (see Fig. 5d—Right).

The most common techniques to encapsulate growth factors and drugs into the carriers are cross-linked polymer hydrogels^34^ and encapsulation in nanoparticles.^153^ For example, Qi et al. synthesized BMP-2 nanocapsules via in-situ polymerization on the surface of BMP-2, using 2-(methacryloyloxy)ethyl phosphorylcholine (MPC) as monomer and MMP as cleavable peptide crosslinker. After the cleavable crosslinkers are degraded in situ by MMP-2 and MPP-9, the growth factor (BMP-2) is released into the ECM to promote the healing of bone fractures.^159^ Zhang et al.^160^ developed an injectable BMSCs-laden hydrogel scaffold with encapsulated rhBMP-2 to promote bone regeneration. When exposed to hydrogen peroxide (H_2_O_2_) and horseradish peroxidase, the scaffold is able to fill defected bones within 15 s and after 14 days promotes BMSCs proliferation and realize osteogenic differentiation.^160^

Some other examples of EMR-based drug delivery applications are the controlled delivery of minocycline hydrochloride activated by a change in ALP levels for the treatment of periodontal disease,^161^ the use of chitosan as a substrate for drug encapsulation endows the membrane with additional antibacterial characteristics,^161^ and the release of deferoxamine triggered by hyaluronidase to avoid bacterial infection and aseptic loosening in titanium implants.^170^

### Summary

Responsive biomaterials can respond to external stimuli—out-body or in-body—such as mechanical forces, electrical or magnetic fields, temperature, pH, and enzymatic changes to trigger a specific response or behavior. Some of these smart materials are able to interact with tissues and cells promoting cell differentiation, proliferation, and eventually increase osseointegration and bone regeneration. Others can be used for the controlled release of drugs, growth factors, or the development of medical devices.

## Immune-modulatory materials for bone regeneration

Immunomodulatory biomaterials are rather recent newcomers to the family of smart biomaterials for bone regeneration.^171^ In the context of this review, immunomodulatory biomaterials can be defined as any biomaterial which has the capability of manipulating the host immune system towards inducing hard tissue repair and/or regeneration of bone either locally or systemically.^171^ Indeed, researchers have begun using biomaterials that activate immunomodulation to “trick” the body into repairing itself. For example, Xue et al.,^172^ demonstrated that GO can be a biomaterial with immunomodulatory capabilities. When cultured with mouse macrophages, GO induced the formation of a beneficial osteo-immunomodulatory environment which caused osteogenic differentiation of BMSCs, stimulated the upregulation of vascular-related receptors in human umbilical vein endothelial cells, and promoted their tube formation in vitro.^172^

Immunomodulatory biomaterials can be categorized into four separate classes based on their methods of, respectively, action and activation. (a) Phenotypic switch of the cells of the immune system (predominantly macrophages) from a destructive inflammatory phenotype to a constructive regenerative phenotype, (b) exogenous surface coatings, (c) innate material properties, and (d) direct activation of stem cell-mediated regeneration.

As a key component of our innate immune system, macrophages are primarily responsible for digesting foreign bacteria, killing cancer cells, and eliminating cellular debris through phagocytosis. Macrophages play an integral role in chronic inflammation as they remove the aged neutrophils; neutrophils are the body´s first defense against bacterial infection and acute inflammation. As wounds progress and the inflammation subsides, macrophages begin to take on the role of mediating wound healing and tissue repair by activating tissue fibroblasts to produce collagen, which in turn is needed for revascularization and re-epithelialization of the damaged tissue. Depending on which stage of its life a macrophage is in and the role it plays, a macrophage can be defined as either a type M1 or type M2 macrophage. The M1 and M2 phenotypes are characterized by different repertoires of, respectively, inflammatory and reparative cytokines. In simple terms, M1 macrophages are the “destructive” bacteria-killing macrophages, which produce inducible nitric oxide and are present during the early phases of wound healing. M2 macrophages are the anti-inflammatory, reparative, “constructive” macrophages, which develop from M1 macrophages, and are responsible e.g., for inducing the production of collagen in the regenerating tissue.

Over the past few years we have significantly increased our insight into the ability of biomaterials to improve bone tissue repair and regeneration modulating by modulating the macrophage phenotype from M1 macrophages to M2 macrophages (macrophage polarization).^173–177^ For example, Wu et al.^175^ developed a practical and economical process that influences the immunomodulation of bone ECM before in vivo transplantation.^175^ For this, the authors produced a modified ECM gel which was loaded with bone-derived filler particles to optimize the immunomodulatory properties of traditional bone ECM.^175^ After 21 days in a rat periodontal model, the modified ECM polarized the macrophages towards the regenerative, anti-inflammatory, constructive M2 macrophage phenotype, leading to enhanced tissue regeneration.^175^

Zhang et al.^155^ used strontium-doped sub-micrometer bioglass (Sr−SBG) as an immunomodulatory biomaterial for bone repair/regeneration.^176^ When used alone, bioglass (BG) has excellent osteoconductive and osteoinductive properties and can induce osteoblast differentiation. However, once strontium was incorporated in the bioglass, the immunomodulatory properties increased and elicited a beneficial effect by inhibiting the pro-inflammatory response of the macrophages. Taken together, the physiological responses induced by Sr−SBG enhanced osteogenesis while inhibiting osteoclastogenesis, meaning more bone was produced during the healing stage, while less was being resorbed by the osteoclasts.

As described above for implants that have been coated with Ta to increase their antibacterial properties, exogenous surface coatings can be utilized to activate immunomodulation in the body, thus triggering improved repair and regeneration. Both metals, such as zinc and copper, as well as nonmetals, like calcium phosphate and calcium silicate, have been used to coat the surfaces of biomaterials to activate immunomodulation.^173,174,178,179^ Another example of a surface coating which has immunomodulatory capabilities is boron incorporated into calcium silicate (B-CS). The B-CS coating decreased the number of M1 macrophages and polarized them to the M2 phenotype.^174^ This was accomplished by limiting the toll-like receptor signaling pathway which resulted in a significant reduction in the production of pro-inflammatory cytokines while causing an increase in anti-inflammatory, pro-reparative cytokines.^174^

Other surface modifications consist of preloading immunomodulatory cytokines, such as interleukin-4 (IL-4), directly into titanium oxide (TiO_2_) nanotubes, and then coating bone implant surfaces with these nanotubes.^180^ IL-4 is one of the cytokines that trigger the polarization of inflammatory M1 macrophages into reparative M2 macrophages.^180^ The IL-4 loaded nanotube coating did indeed stimulate macrophage polarization from the M1 phenotype to M2 phenotype.^180^

Similar to the effects of exogenous surface coatings, the innate surface topography of an implant can affect how the body reacts to a given material. Specifically, some surface topographies can exert immunomodulatory effects by polarizing macrophages towards the M2 phenotype to aid in bone regeneration and increasing the expression of anti-inflammatory cytokines to decrease inflammation, and promote osteoblast differentiation.^181–185^ For example, in analyzing the immunomodulatory properties of the hierarchical macropore/nanosurface topography of surfaces that had been coated with titanium (Ti) via plasma spray, Pan et al.^186^ found preferential macrophage polarization towards the M2 phenotype as well as decreasing levels of inflammatory genes and increased expression of anti-inflammatory genes.^186^ In analyzing the mechanisms responsible for this macrophage polarization, Pan et al.^186^ hypothesized that the concomitant decrease in inflammatory genes and increase in anti-inflammatory genes is regulated by the cytoskeletal tension, which is induced by altered cell shape on the hierarchical Ti surface.^186^ By growing on the nano-modified titanium surface, the shape of the macrophages is changed into one which will activate a pro-reparative M2 phenotype and thus enhance bone regeneration and healing via macrophage phenotype manipulation.^186^

Stem cells play a key conceptual and practical role in tissue engineering and regenerative medicine, due in part to their pluripotency, but also due to their immunomodulatory properties. The environment that stem cells are placed in, may contribute to their ability to promote regeneration in the surrounding area. For example, in the context of bone regeneration, addition of β-TCP to enhance the osteogenic differentiation of BMSCs or treating stem cells derived from exfoliated deciduous teeth with acetylsalicylic acid will significantly improve osteogenic differentiation and enhance their immunomodulatory competence by upregulating the production of telomerase reverse transcriptase.^187^

Another method of controlling the environment of a stem cell is placing them on a modified synthetic biomaterial scaffold. Silicified collagen scaffolds (SCS) are semi-synthetic scaffolds made from collagen matrices permeated with intrafibrillar amorphous silica.^188^ When seeded with BMSCs, these SCS-BMSC constructs promote in situ bone growth and regeneration as well as angiogenesis by modulating the numbers and immunocompetence of tissue-resident monocytes.^188^ Specifically, as the silica in the scaffolds breaks down it releases silicic acid which has been shown to stimulate the differentiation of monocytes into macrophages.^188^ The silicic acid-differentiated macrophages exhibit an increased expression of several reparative cytokines and growth factors, like SDF-1α, TGF-β1, VEGFa, and PDGF-BB, further promoting the differentiation of BMSCs and endothelial progenitor cells and enhancing neovascularization.^188^

### Summary

Multiple mechanisms promote the interactions between bone tissue and the immune system. The detailed knowledge of these interactions will provide a solid scientific basis for the development of a new category of smart biomaterials for bone regeneration called immune-modulatory materials. These materials are capable of manipulating and regulate the host immune system to advance key regenerative effects such as initiate tissue repair, decrease inflammation, or promote osteoblast differentiation. The use of such immune-modulatory smart materials might help to overcome complications in bone healing and bone-related diseases and will allow the development of more effective and safer therapeutics and more effective regenerative engineering approaches.

## Translation: from basic research to bedside

Recent advances in basic research has led to an explosion of knowledge about the mechanisms underlying many diseases and physiological processes. There is an abundance of new biomedical technologies and products originating from academia.^189^ For example, the number of patents granted annually by the United States Patent and Trademark Office to U.S. university continues to increase rapidly, more than doubling between 2008 and 2016, reaching more than 6 600 in 2016.^190^ University applications for U.S. patents also increased over time with 13 389 filings in 2015.^190^ The quest for translational application of academic research is also reflected in the number of startups that have been spun out of academic institutions. The number of business startups from university technology transfer reached 950 in 2015 after showing consistent growth after 2012, with roughly 100 startups being created every year.^190,191^

On the other side, the translation of these biomedical technologies to the clinic has proven to be challenging. According to the National Institutes of Health, approx. 90% of “translational” academic research projects are never tested in humans^192^ and less than 5% of life science scientific discoveries originated in academia will successfully transition into a change in clinical practice, new medications, diagnostics, or devices.^193^ There is a significant mismatch between the number of biomedical technologies produced by academia and what is actually delivered to clinics and patients.

On the other side, the market demands and clinical needs for new technologies continue to rise. A recent report on global market predictions indicates that the biomaterials market will grow from USD105 billion in 2019 to USD207 billion by 2024 at a compound annual growth rate of 14.5%.^194^ The biomaterials market is segmented by the material type (e.g, ceramic, metallic, natural, polymeric, composites) and according to the applications (e.g. dental, cardiovascular, orthopedic, wound healing, neurology, plastic surgery, tissue engineering). Despite the widespread use of biomaterials in medicine with encouraging marketing forecasts, there are still many major challenges for the safe and effective translation of these technologies in the clinic.^30,195^

There are several recognized roadblocks hindering the translation of basic scientific knowledge to real benefits.^196–199^ Academic culture includes the gap between academic reality and industry expectations, the lack of incentive, a misalignment between translational objectives and the academic reward system, and the lack of an industry network. Translating preclinical academic research to clinical application is particularly challenging, time-consuming, and expensive. As a result, many promising biomaterials technologies that show clear efficacy and safety in the preclinical setting fail to reach the market.^200^

To overcome the “valley of death” and increase the chances for translational success of biomaterials, several strategies have been proposed in the three main areas: academic, regulatory, and federal leadership.^201–204^ Academic leadership includes creating an environment that is conducive to the first steps towards translation, i.e., identifying clinical needs, creating multidisciplinary teams, partnerships with technology transfer offices, funding to de-risk technologies, relations with investors, modifying the academic promotion system, and establishing local academic translational centers/hubs. Regulatory leadership includes lessening of governmental oversight and more flexible approaches toward required animal studies for preclinical validation. Industry leadership includes providing economic incentives, creating industrial partnerships, and sponsoring educational and mentorship programs.

### Summary

In this section, we identified numerous roadblocks that hitherto thwarted the translation of biomedical investigations into the final clinical applications. There is a need for well-orchestrated interactions between academia, regulatory authorities, and industry, the three major players in translating these technologies to help fill the dwindling pipelines of translational research, so that the patients can actually benefit from recent scientific discoveries and technological progress.

## Summary and outlook

While naturally occurring materials have occasionally been used for medical purposes for thousands of years, the last 100 years have witnessed a transformative role of synthetic/engineered smart biomaterials, from inert gem stones used as a placeholder for a lost teeth to complex autonomous materials that are capable of detecting and reacting to different environmental stimuli. The varying degrees of “smartness” that a biomaterial is endowed with depends on several factors such as external stimuli and inherent material properties and can be tuned to provide different levels of benefits. We anticipate that biomaterials that have immunomodulatory capabilities will be in high demand in the coming decades as clinicians and scientists learn to understand their capabilities. These immunomodulatory biomaterials will become the next generation of smarter, more adaptive biomaterials because these materials are able to manipulate the immune system of the host to create an environment in which healing, regeneration, and repair is promoted and regulated. Like traditional biomaterials, they can be made from many different substrates, however material composition and fabrication methods must be taken into special consideration in order to create most permissive, most regenerative environment for a chosen application. Combining diverse fabrication methods from areas of research not traditionally associated with biomaterials or medicine has led to the development of many distinct technologies, such as 3D and 4D bioprinting, electronic beam melting, and robocasting. These new technologies produce tailor-made biomaterials at a level that was unachievable with previous manufacturing methods. Continuing the interdisciplinary collaborations of researchers from different fields, not just medicine and engineering, will advance the field of biomaterials and help tackle the challenges that lie ahead. Once the research and development of smart biomaterials in the lab is complete, a concerted effort must be made to transition these materials from the laboratory into clinical practice so that the general population many benefit from the materials’ unprecedented capacities to heal, repair, and regenerate.
